# Genome-wide DNA methylation analysis of pseudohypoparathyroidism patients with GNAS imprinting defects

**DOI:** 10.1186/s13148-016-0175-8

**Published:** 2016-01-26

**Authors:** Anne Rochtus, Alejandro Martin-Trujillo, Benedetta Izzi, Francesca Elli, Intza Garin, Agnes Linglart, Giovanna Mantovani, Guiomar Perez de Nanclares, Suzanne Thiele, Brigitte Decallonne, Chris Van Geet, David Monk, Kathleen Freson

**Affiliations:** Department of Cardiovascular Sciences, Center for Molecular and Vascular Biology, University of Leuven, Campus Gasthuisberg, O&N1, Herestraat 49, Box 911, 3000 Leuven, Belgium; Department of Pediatrics, University Hospitals Leuven, 3000 Leuven, Belgium; Laboratory of Genomic Imprinting and Cancer, IDIBELL, 08908 Barcelona, Spain; Fondazione IRCCS Ca’ Granda Ospedale Maggiore Policlinico, Endocrinology and Diabetology Unit, Department of Clinical Sciences and Community Health, University of Milan, 20122, Milan, Italy; Molecular (Epi)Genetics Laboratory, BioAraba National Health Institute, Hospital Universitario Araba-Txagorritxu, 01009 Vitoria-Gasteiz, Spain; Department of Pediatric Endocrinology and Diabetology for Children, APHP, Bicêtre Paris Sud, 94275 Le Kremlin Bicêtre, France; Reference Center for Rare Disorders of the Mineral Metabolism and Plateforme d’Expertise Paris Sud, APHP, 94275 Le Kremlin Bicêtre, France; Division of Experimental Paediatric Endocrinology and Diabetes, Department of Paediatrics, University of Luebeck, 23560 Luebeck, Germany; Department of Clinical and Experimental Endocrinology, University of Leuven, 3000 Leuven, Belgium

**Keywords:** Pseudohypoparathyroidism, *GNAS*, DNA methylation, Imprinting disorders

## Abstract

**Background:**

Pseudohypoparathyroidism (PHP) is caused by (epi)genetic defects in the imprinted *GNAS* cluster. Current classification of PHP patients is hampered by clinical and molecular diagnostic overlaps. The European Consortium for the study of PHP designed a genome-wide methylation study to improve molecular diagnosis.

**Methods:**

The HumanMethylation 450K BeadChip was used to analyze genome-wide methylation in 24 PHP patients with parathyroid hormone resistance and 20 age- and gender-matched controls. Patients were previously diagnosed with *GNAS*-specific differentially methylated regions (DMRs) and include 6 patients with known *STX16* deletion (PHP^Δstx16^) and 18 without deletion (PHP^neg^).

**Results:**

The array demonstrated that PHP patients do not show DNA methylation differences at the whole-genome level. Unsupervised clustering of *GNAS*-specific DMRs divides PHP^Δstx16^ versus PHP^neg^ patients. Interestingly, in contrast to the notion that all PHP patients share methylation defects in the *A/B* DMR while only PHP^Δstx16^ patients have normal *NESP*, *GNAS-AS1* and *XL* methylation, we found a novel DMR (named *GNAS-AS2*) in the *GNAS-AS1* region that is significantly different in both PHP^Δstx16^ and PHP^neg^, as validated by Sequenom EpiTYPER in a larger PHP cohort. The analysis of 58 DMRs revealed that 8/18 PHP^neg^ and 1/6 PHP^Δstx16^ patients have multi-locus methylation defects. Validation was performed for *FANCC* and *SVOPL* DMRs.

**Conclusions:**

This is the first genome-wide methylation study for PHP patients that confirmed that *GNAS* is the most significant DMR, and the presence of *STX16* deletion divides PHP patients in two groups. Moreover, a novel *GNAS-AS2* DMR affects all PHP patients, and PHP patients seem sensitive to multi-locus methylation defects.

**Electronic supplementary material:**

The online version of this article (doi:10.1186/s13148-016-0175-8) contains supplementary material, which is available to authorized users.

## Background

**Fig. 1:**
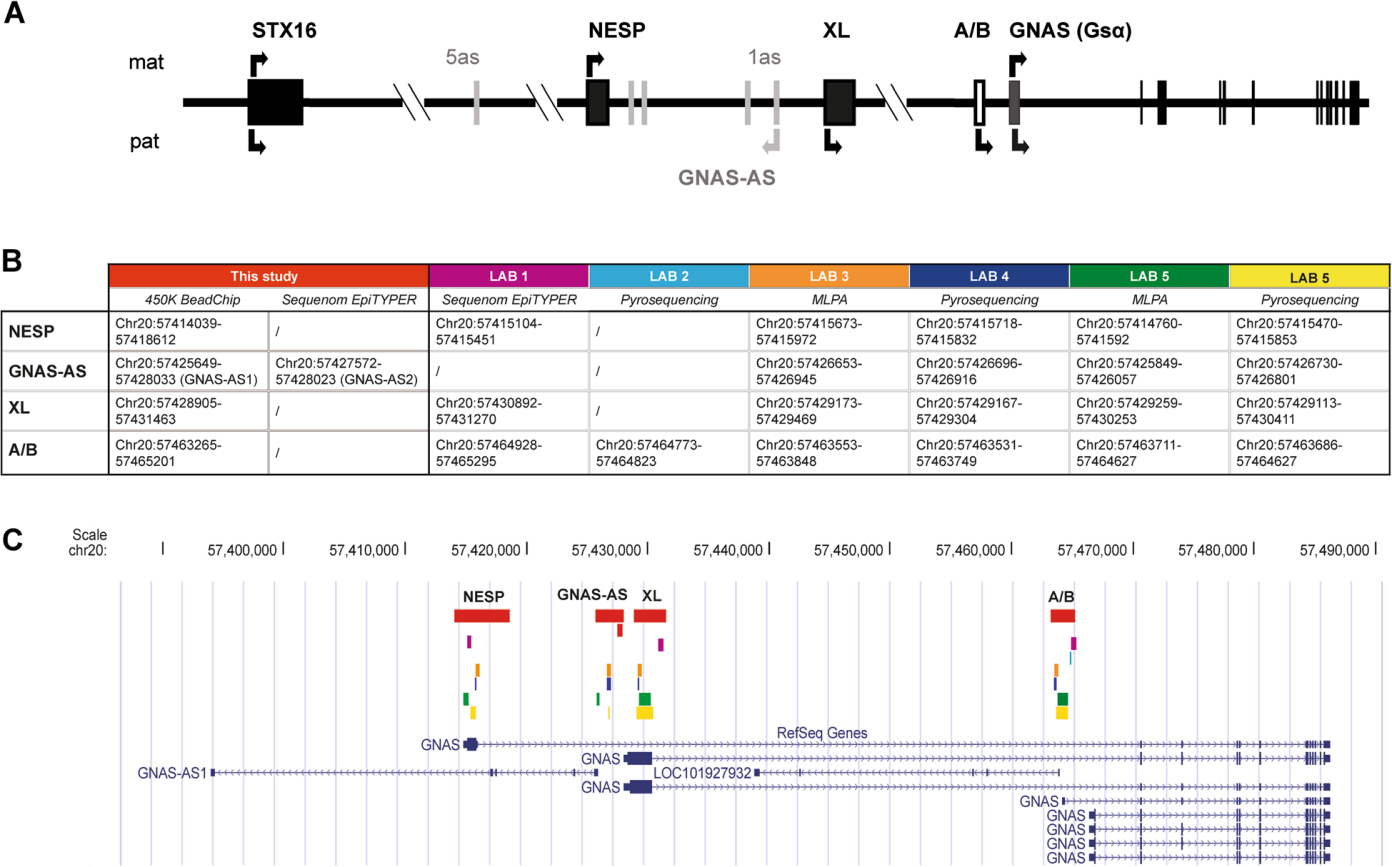
Human *GNAS* cluster. **a** Schematic diagram of the human *GNAS* cluster with DMRs *A/B*, *XL*, *GNAS-AS*, and *NESP* and the upstream *STX16* gene region. The *arrows* show initiation and direction of transcription for maternal (*mat*)- and paternal (*pat*)-derived transcripts. **b** Overview of the different *GNAS* methylation studies that were performed for 24 PHP patients in this study using the HM450k and Sequenom EpiYTPER and previously, by five different European centers using the specified techniques and loci. **c** Detailed schematic representation of the four *GNAS* DMRs investigated by the different European laboratories and the consortium. Nucleotide positions are indicated according to NCBI build 37/hg19

Genomic imprinting is a parent-of-origin dependent gene expression that is essential for mammalian development. A mechanism underlying allele-specific expression is DNA methylation. The addition of a methyl group to DNA cytosine nucleotides at CpG sites can occur in an allele-specific manner, and allele-specific methylation in imprint control regions (referred to as differentially methylated regions (DMRs)) is associated with parent-of-origin dependent gene expression. Imprinting disorders are a group of rare diseases affecting growth, development, and metabolism that are associated with (epi)genetic disruption of imprinting genes [1]. Pseudohypoparathyroidism (PHP) is a rare endocrine disorder that can be caused by genetic or epigenetic alterations in the imprinted cluster *GNAS* localized on chromosome 20q13.3 [2]. The human *GNAS* locus harbors four DMRs encompassing the promoters of four alternative transcripts: exon A/B (*GNAS-A/B*: TSS DMR = ***A/B***), GNAS antisense (*GNAS-AS1*: TSS DMR = ***AS1***), extra-large stimulatory G protein (*GNAS-XL*: Ex1 DMR = ***XL***), and neuroendocrine secretory protein 55 (*GNAS-NESP*: TSS DMR = ***NESP***) (Fig. 1a) [3]. PHP type I (PHP1A and PHP1B) patients are characterized by end-organ resistance to the action of the parathyroid hormone (PTH), which leads to hypocalcemia, hyperphosphatemia, and elevated levels of PTH in the absence of vitamin D deficiency. They often also have thyroid-stimulating hormone (TSH) resistance [2, 3]. PHP1A patients have in addition to hormone resistance clinical features collectively referred to as Albright’s hereditary osteodystrophy (AHO) that include brachydactyly, short stature, and round face; they may also present with obesity, subcutaneous ossifications, mental retardation, and behavior problems [4, 5]. PHP1A is caused by heterozygous maternally inherited inactivating mutations in the coding sequence of Gsα (exons 1 to 13 of *GNAS*) [2, 3, 6]. On the other hand, paternally inherited inactivating mutations lead to pseudopseudohypoparathyroidism (PPHP), characterized by AHO features but without hormone resistance [2, 3, 6]. Most patients affected with the PHP1B form of the disease exhibit mainly PTH resistance and subclinical TSH resistance and do not have AHO features. In some cases however, typical brachydactyly, severe obesity [7], and congenital hypothyroidism [8, 9] have been described in PHP1B patients highlighting the overlap between PHP1A and PHP1B. All PHP1B patients have methylation abnormalities in the *GNAS* cluster with loss of imprinting at the *A/B* DMR [6]. The familial form of PHP1B (often referred to as autosomal dominant AD-PHP1B) is typically associated with microdeletions in the *STX16* region located upstream of the *GNAS* cluster [10–13] and less frequently with deletion removing the *NESP* DMR [14]. On the other hand, sporadic PHP1B patients have broad *GNAS* methylation defects that involve all four *GNAS* DMRs without a known underlying genetic cause [2, 3, 6].

However, from recent data, it becomes clear this original PHP classification is no longer accurate for the following four reasons: (i) broad *GNAS* methylation defects were also found for PHP1B patients with an AHO phenotype [7, 15–17], (ii) the degree of the methylation defect seems not to correlate with the disease severity [18], (iii) reduced Gsα activity is no longer exclusive for PHP1A with inactivating mutations but recently also was described for PHP patients with epigenetic defects [19, 20], and (iv) partial *GNAS* methylation defects have been described [21]. Moreover, as shown for other imprinting disorders, such as Silver-Russell or Beckwith-Wiedemann syndrome, also some PHP1B patients were shown to have multi-locus imprinting defects as studied by a targeted approach that comprised known imprinted loci [22–24]. Therefore, there is a strong need to improve the current classification of PHP patients that might be feasible based on more detailed (epi)genetic or clinical data using larger patient cohorts analyzed with more powerful techniques. The European Consortium for the study of PHP (EuroPHP) designed the present genome-wide methylation study to gain insight in epigenetic profiles that might improve an epigenetic-based classification. We have analyzed the methylomes of 24 previously diagnosed PHP patients with *GNAS* epigenetic defects recruited from five European centers. This sample set includes 6 patients with the *STX16* 3-kb recurrent microdeletion (PHP^Δstx16^) [11] and 18 without any identified deletion within the *GNAS* locus (PHP^neg^). The following analyses were performed: (i) changes in DNA methylation at the genome-wide level, (ii) unsupervised cluster analysis for the *GNAS* locus and comparisons with previous *GNAS* methylation data used for PHP diagnosis, and (iii) detailed DNA quantification analysis for other imprinted DMRs. DNA methylation data were validated with the Sequenom EpiTYPER for additional PHP patients with PTH resistance and proven epigenetic defects.

## Results

### Genome-wide DNA methylation analysis for PHP patients

**Fig. 2:**
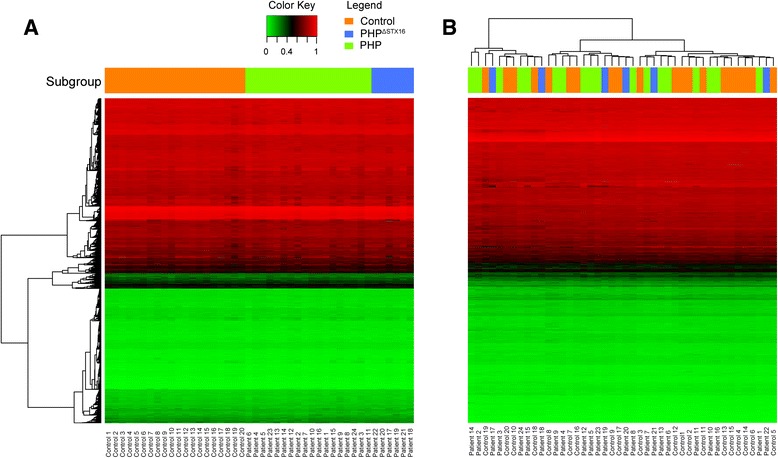
Whole-genome methylation in PHP patients versus controls. Methylation values are extracted from data obtained with the HM450k. **a** Clustering according to different subgroups. **b** Unsupervised hierarchical clustering analysis of the subgroups. *Control* controls, *PHP* ^*ΔSTX16*^ patients with underlying *STX16* deletion, *PHP* patients without known genetic deletion. Heatmaps represent 3575 randomly selected CpGs (1 % total CpGs)

The Illumina Infinium HumanMethylation 450K BeadChip (HM450k) was used to determine genome-wide DNA methylation profiles in 24 PHP patients and 20 age- and gender-matched healthy controls. Table 1 presents the clinical characteristics of the PHP patients that all have PTH resistance and hypocalcemia. Mild AHO features were only present in patients 8, 10, 13, 15, and 20. All 24 PHP patients were previously diagnosed with a *GNAS* epigenetic defect using different methodologies (Fig. 1b, c), and methylation values for the different *GNAS* DMRs obtained by these assays are shown in Table 1. We have included 6 PHP patients with a deletion of the *STX16* region upstream of *GNAS* and hypomethylation of the *A/B* DMR only (referred to as PHP^Δstx16^) and 18 PHP patients without the deletion and full or partial methylation defects in *A/B*, *XL*, *AS1*, and *NESP* DMRs (referred to as PHP^neg^) (Table 1). Hierarchical cluster analysis of all CpGs according to the different subtypes PHP^Δstx16^, PHP^neg^, or control (Fig. 2a) and unsupervised hierarchical clustering analysis of the data (Fig. 2b) showed that the samples are clustered irrespective of the subgroup, suggesting that there are no subgroup differences at whole-genome level. To further exclude a global methylation defect in PHP patients, we also analyzed the methylation of DNA repetitive elements (*LINE-1* and *LINE-2*) that again showed no differences between patients and controls (data not shown).

**Table 1:** Clinical and molecular characteristics of the PHP patients enrolled in the genome-wide DNA methylation study

1. Clinical characteristics									2. Initial diagnostic screening							3. HM450k				
Patient	Gender	PTH-res	TSH-res	Ca ↓	P ↑	OS	BD	Additional features	STX16	LAB	Label	NESP	AS	XL	A/B	NESP	AS1	AS2	XL	A/B
1	F	Yes	Yes	Yes	Yes	No	No	Two café-au-lait spots, autoimmune thyroiditis		4	Full PHP1B—broad	94	7	27	3	90^**^	10^**^	8^**^	34^*^	13^**^
2^*^	M	Yes	Yes	Yes	Yes	No	No			3	Full PHP1B—broad	80	7	8	2	89^**^	10^**^	8^**^	6^**^	12^**^
3	M	Yes		Yes	Yes	No	No	Von Willebrand disease		5	Full PHP1B—broad	88	4	6	0	88^**^	10^**^	8^**^	7^**^	13^**^
4^*^	M	Yes	No	Yes	Yes	No	No			1	Full PHP1B—broad	92		8	7	89^**^	10^**^	8^**^	7^**^	13^**^
5^*^	M	Yes	No	Yes	Yes	No	No			3	Full PHP1B—broad	82	7	8	2	89^**^	9^**^	8^**^	7^**^	12^**^
6	F	Yes	No	Yes	Yes	No	No			1	Full PHP1B—broad	92		10	9	89^**^	9^**^	7^**^	5^**^	12^**^
7	M	Yes	No	Yes	Yes	No	No	Bilateral cryptorchidism, osteopenia of hands, behavior problems		4	Full PHP1B—broad	95	5	4	3	89^**^	9^**^	8^**^	5^**^	12^**^
8^*^	F	Yes		Yes	No	No	Yes			5	Full PHP1B—broad	90	3	4	0	89^**^	10^**^	8^**^	6^**^	12^**^
9	F	Yes	Yes	Yes	No		No	Enchondromatosis		5	Partial PHP1B—broad	83	23	34	33	63	27	23	24^*^	35
10	F	Yes	No	Yes	Yes	No	Yes	Exostosis		4	Partial PHP1B—broad	84	20	24	13	75^*^	22^*^	16^**^	33^*^	31
11	F	Yes								5	Partial PHP1B—broad	80	10	27	14	78^*^	23^*^	19^*^	34^*^	28
12^*^	M	Yes	Yes	Yes	No	No	No	Severe hypertension with organ damage, hypercalciuria		3	Full PHP1B—broad	77	8	8	3	75^*^	12^**^	8^**^	7^**^	17^**^
13^*^	F	Yes	Yes	Yes	Yes	No	Yes			3	Partial PHP1A—broad	76	8	7	5	73^*^	15^**^	9^**^	5^**^	21^*^
14^*^	M	Yes	Yes	Yes	No	No	No			3	Partial PHP1B—broad	80	10	19	29	76^*^	19^**^	13^**^	30^*^	21^*^
15	F	Yes	Yes	Yes	Yes	Yes	No	Langerhans cell histiocytosis, neonatal hydrocephaly, sessile exostosis		4	Partial PHP1A—broad	86	18	18	7	81^**^	18^**^	15^**^	32^*^	21^*^
16	F	Yes	Yes	Yes	Yes	No	No	Fahr’s syndrome (calcifications of basal ganglia), autoimmune thyroiditis		4	Partial PHP1B—broad	91	12	30	7	85^**^	17^**^	16^**^	43	21^*^
17	F	Yes	No	Yes	No	No	No		ΔSTX16	3	PHP1B—A/B only	42	36	44	6	46	36	16^**^	45	14^**^
18^*^	F	Yes	Yes	No	No				ΔSTX16	2	PHP1B—A/B only	-	-	-	4	50	37	14^**^	47	14^**^
19	M	Yes	Yes	Yes	Yes	Yes	No	Calcifications of basal ganglia	ΔSTX16	4	PHP1B—A/B only	49	42	52	3	50	41	18^**^	48	15^**^
20	F	Yes	No	Yes	Yes	No	Yes		ΔSTX16	1	PHP1B—A/B only	39	-	36	10	50	40	16^**^	50	15^**^
21	M	Yes	Yes	No	Yes				ΔSTX16	2	PHP1B—A/B only	-	-	-	4	52	41	16^**^	51	13^**^
22	M	Yes	No	Yes	No	No	No		ΔSTX16	1	PHP1B—A/B only	29	-	38	14	53	42	14^**^	51	14^**^
23^*^	F	Yes	No	Yes	Yes	No	No			3	Partial PHP1B—broad	54	26	30	7	55	36	26	40	41
24	M	Yes	No	Yes	Yes	No	No	Severe osteopenia without ossifications		5	Partial PHP1B—broad	70	35	46	44	57	39	34	41	43
																50 ± 7	46 ± 7	39 ± 7	50 ± 4	51 ± 9

From left to right: (1) clinical characteristics; (2) mean methylation values of *GNAS* studies that were performed previously by five different European centers as described in Fig. 1; and (3) mean methylation values of *GNAS* investigated with HM450k. Ordering of the patients according to unsupervised hierarchical clustering of *GNAS* methylation investigated with HM450k. Patients* have methylation changes at imprinted genes other than *GNAS*. Methylation values are presented as percentage. Mean ± SD of the 20 controls from the HM450k array is shown below the HM450k columns

*BD* brachydactyly, *Ca* calcium, *F* female, *LAB* laboratory of initial diagnostic screening, annotation as shown in Fig. 1, *M* male, *OS* heterotopic ossifications, *P* phosphate, *PTH-res* parathyroid hormone resistance, *TSH-res* thyroid-stimulating hormone resistance, *ΔSTX16* patients with underlying *STX16* deletion

^*^Methylation < or >3SD outside the mean of the controls

^**^Methylation < or >3SD outside the mean of the controls and <0.20 or >0.80 absolute methylation

### GNAS methylation analysis based on HM450k data for PHP patients

**Fig. 3:**
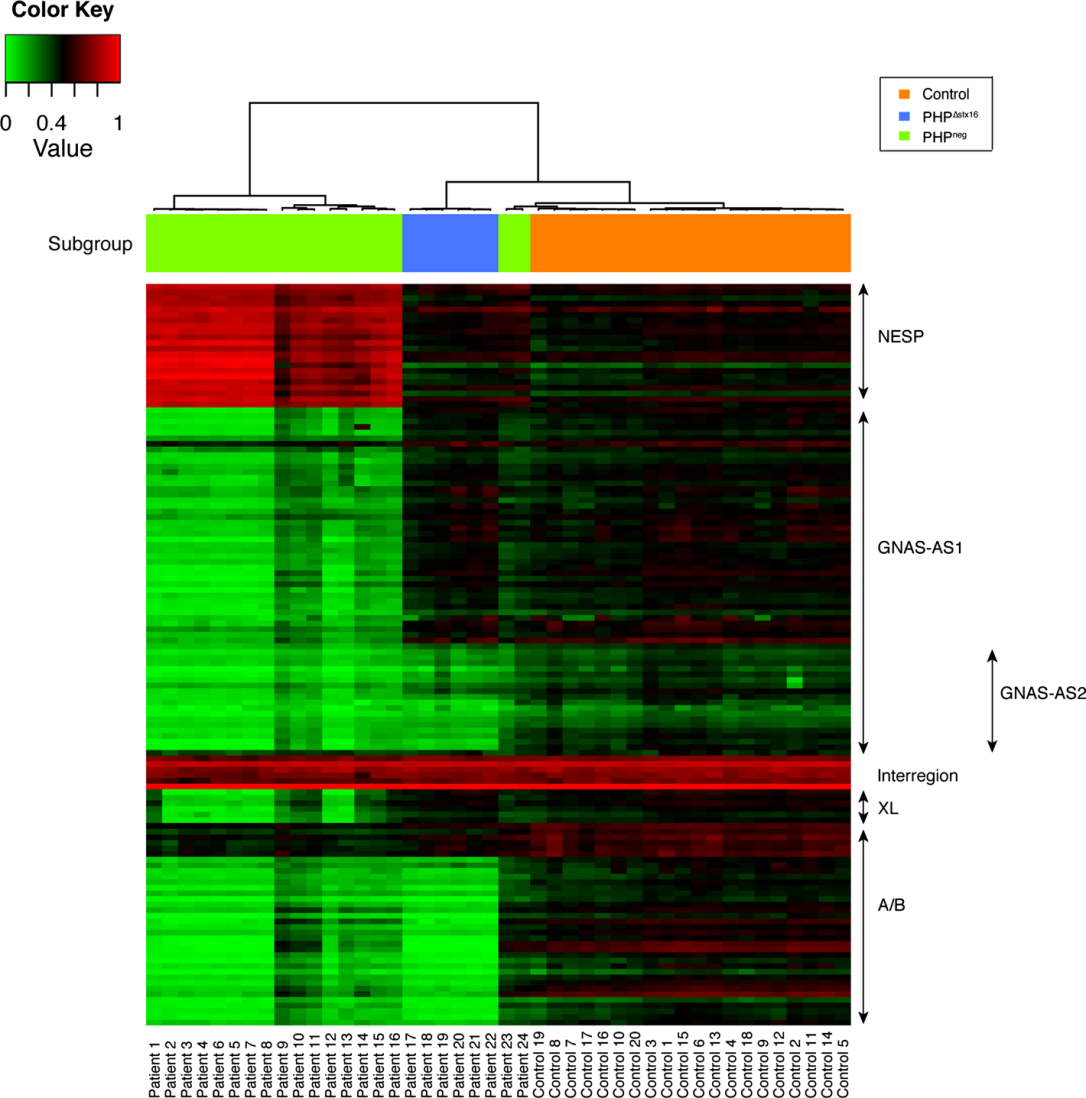
Unsupervised hierarchical clustering of *GNAS* methylation in PHP patients. Methylation values of the individual CpGs of the four *GNAS* DMRs are extracted from data obtained with the HM450k. *Green* and *red* represent 0 and 1 methylation, respectively. The *arrows* show the different *GNAS* transcripts

The methylation values for all CpGs located within the *GNA*S cluster were extracted from data obtained from the genome-wide methylation analysis (Additional file 1: Table S1). The HM450k contains probes mapping to four regions that cover the four *GNAS* DMRs as specified by red boxes in Fig. 1c (referred to as *A/B*, *XL*, *AS1*, and *NESP*). These regions are larger but overlap with the regions that were previously investigated by five European centers that have used different technologies including MS-MLPA, Sequenom EpiTYPER, or pyrosequencing to quantify the methylation of the *GNAS* DMRs for the 24 PHP patients (see Fig. 1c for the location of the studied regions and methodologies and Table 1 for the results for each patient). Unsupervised hierarchical cluster analysis of *GNAS* methylation data obtained from the HM450k for the 24 PHP patients divided these patients in 2 groups according to the presence/absence of *STX16* deletion (PHP^Δstx16^ versus PHP^neg^) (Fig. 3). As known in literature [2, 3] and confirming the previous diagnosis that was made by the 5 laboratories, PHP^Δstx16^ only showed hypomethylation of DMR *A/B* while PHP^neg^ patients had abnormal methylation for all 4 *GNAS* DMRs. However, 2 PHP^neg^ patients (23 and 24) were clustered closer to the healthy control population having normal methylation values for the all *GNAS* DMRs (Table 1 and Fig. 3). These patients were previously identified with a “partial” *GNAS* methylation defect using MS-MLPA and pyrosequencing respectively. After analyzing the separate CpGs from the HM450k, no significant differences were found compared to the control population (separated CpGs are visualized in Fig. 3). At the individual probe level, we noticed that both patients were mosaic and patient 23 was slightly more hypomethylated than patient 24.

**Fig. 4:**
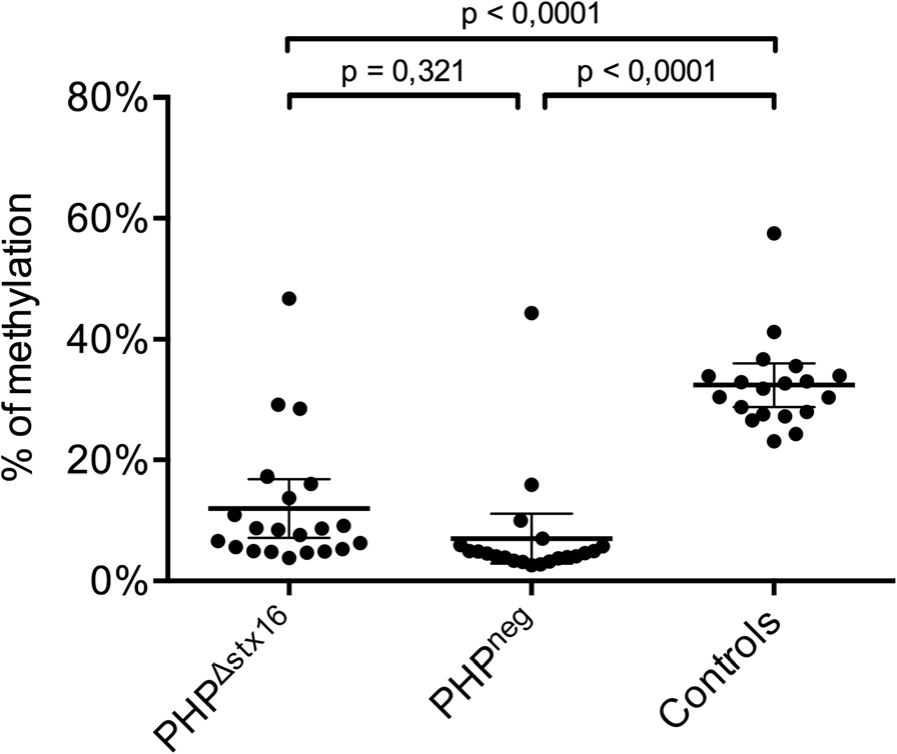
Scatter plot of *NESP-AS2* methylation in PHP patients and controls. Methylation values are obtained with the Sequenom EpiTYPER. *Horizontal bars* indicate the mean and SD of the group. PHP patients are separated according to underlying *STX16* deletion

Interestingly, a smaller region we referred to as *AS2* within the *AS1* amplicon had significantly lower methylation values for both PHP^Δstx16^ and PHP^neg^ patients compared to the controls (Fig. 3 and Table 1). This *AS2* region does not overlap with amplicons that were previously studied for the *AS* region by MS-MLPA, Sequenom EpiTYPER, or pyrosequencing by the different laboratories (Fig. 1b, c and Additional file 2: Figure S1). The *AS2* region is separated from *XL* by a hypermethylated region (Fig. 3). To confirm *AS2* hypomethylation, a validation study was developed using the Sequenom EpiTYPER to quantify methylation in the *AS2* region (Additional file 2: Figure S1) in 42 PHP patients with PTH resistance that were previously diagnosed with a *GNAS* epimutation and 20 age- and gender-matched healthy controls. These 42 PHP patients include 20 PHP^Δstx16^ and 22 PHP^neg^ patients, of which 3 patients in each group were also analyzed by HM450k array (Additional file 1: Table S2). The 22 PHP^neg^ comprise 20 PHP1B with full or partial broad *GNAS* methylation defect and 2 PHP1B patients with isolated A/B methylation defect but having no *STX16* deletion (Additional file 1: Table S3). Significant *AS2* hypomethylation (*P* value <0.0001) was detected for both PHP^Δstx16^ and PHP^neg^ patient groups compared to controls (Fig. 4 and Additional file 1: Table S3). No significant difference in *AS2* methylation was found between PHP^Δstx16^ and PHP^neg^ patients with mean methylation values of 10 % (95 % confidence interval (CI) 7–13 %) versus 8 % (95 % CI 5–11 %), respectively. This suggests that the imprinting defect for this interval is not determined by the *STX16* deletion and is specific for PHP1B. To investigate if *AS2* hypomethylation changed *XL* expression, we performed *GNAS* versus *XL* messenger RNA (mRNA) expression studies using total blood mRNA from a PHP^neg^ patient and control heterozygous for *GNAS* SNP (rs7121), but we could not detect *XL* expression in blood cells.

### Methylation studies of other imprinted genes for PHP patients

**Fig. 5:**
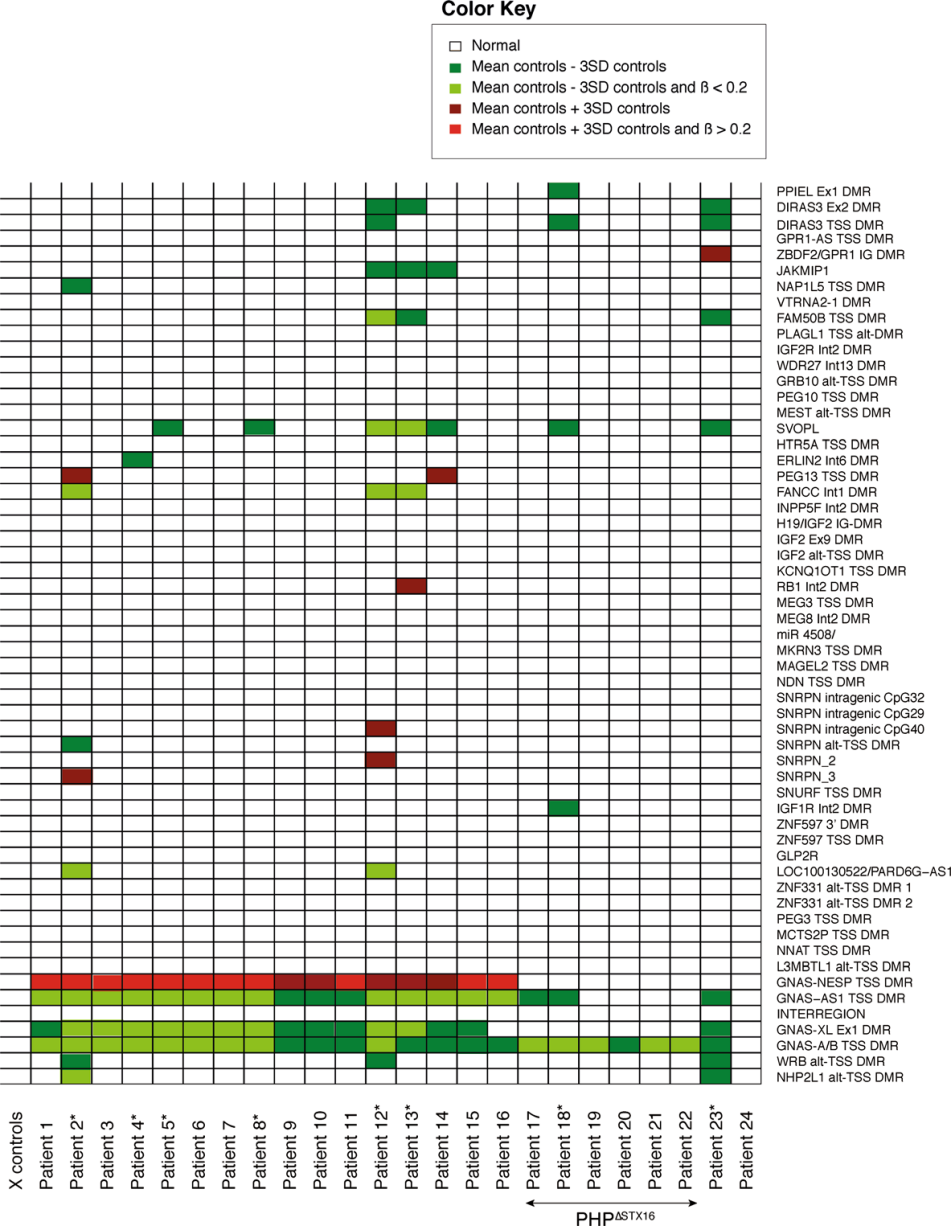
Heatmap showing methylation of known imprinted genes in PHP patients. Methylation values of the genes are extracted from data obtained with the HM450k. *Dark green* and *red* respresent −3SD and +3SD, respectively, of the mean as determined in 20 controls. *Lighter green* and *red* represent additional cut-offs for absolute methylation <0.20 and >0.80. Patients* have multi-locus methylation abnormalities. *PHP* ^*ΔSTX16*^ patients with underlying *STX16* deletion

As multi-locus abnormalities have also been described for PHP1B patients [23], we analyzed the methylation values for all CpGs located within 58 imprinted DMRs for humans that are covered by the HM450k (Additional file 1: Table S4 and S5). In addition to methylation abnormalities for the 4 *GNAS* DMRs, we found multi-locus methylation defects in 8 PHP^neg^ and 1 PHP^Δstx16^ patients (Fig. 5). Abnormal methylation was found for 19 of the 58 human imprinted DMRs, and this included significant hypomethylation for *PPIEL*:Ex1-DMR, *DIRAS3*:Ex2-DMR, *DIRAS3*:TSS-DMR, *JAKMIP1*, *NAP1L5*:TSS-DMR, *FAM50B*:TSS-DMR, *SVOPL*, *FANCC*:Int1-DMR, *SNRPN*:alt-TSS-DMR, *IGF1R*:Int2-DMR, *LOC100130522/PARD6G-AS1*, *WRB*:alt-TSS-DMR, and *NHP2L1*:alt-TSS-DMR and significant hypermethylation for *ZBDF2*/*GPR1*:IG-DMR, *PEG13*:TSS-DMR, *RB1*:Int2-DMR, *SNRPN* intragenic *CpG40*, *SNRPN*_*2*, and *SNRPN*_*3. ZBDF2*/*GPR1*:IG-DMR hypermethylation is likely due to *GPR1-AS*:TSS-DMR hypomethylation. The *GPR1-AS*:TSS-DMR is not included in our screening, as it acquires immediate biparental methylation following implantation; therefore, we can only infer loss of methylation due to hypermethylation of *ZBDF2* [25]. The clinical phenotype of the patients with multi-locus methylation abnormalities was not different from the PHP patients with *GNAS*-specific epimutations (Table 1).

**Fig. 6:**
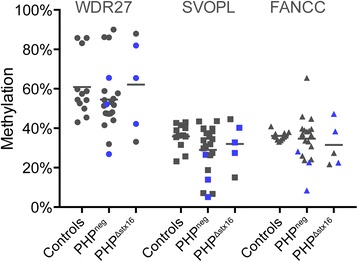
Scatter plot of *FANCC* and *SVOPL* methylations in PHP patients and controls. Methylation values are obtained with the Sequenom EpiTYPER. *Bar* indicates the mean of the group. PHP patients are separated according to underlying *STX16* deletion. Patients that were investigated with the HM450k are indicated in *blue*

To confirm the findings of multi-locus abnormalities, we performed a validation study using the Sequenom EpiTYPER for 26 PHP patients and 12 healthy controls. We have selected *FANCC* and *SVOPL* as hypomethylation was seen in 3 and 7 PHP patients, respectively. We also validated the methylation of *WDR27* amplicon as negative control. The 26 PHP patients included 5 PHP^Δstx16^ and 21 PHP^neg^ patients; 3 patients of each group were also analyzed by HM450k (Additional file 1: Table S2). Eight patients had multi-locus methylation abnormalities (Fig. 6 and Additional file 1: Table S6). The most significant methylation difference was found for *SVOPL* in three PHP^neg^ patients.

## Discussion

Genome-wide studies to identify genetic alterations have successfully entered the field of rare diseases [26]. Similar attempts to elucidate the epigenome are expected to improve our limited knowledge about imprinting disorders (IDs). There has been a revolution in DNA methylation analysis technology over the past decade as genome-wide and entire methylomes can be characterized at single-base-pair resolution [27]. Their implementation to study IDs has only just started. A custom Illumina GoldenGate array targeting 27 imprinted DMRs has been used to study 65 patients with an ID (that include Beckwith-Wiedemann syndrome (BWS), transient neonatal diabetes mellitus (TNDM), PHP1B, Silver-Russell syndrome (SRS), Angelman syndrome (AS), and Prader-Willi syndrome (PWS)) and found multi-locus hypomethylation in patients with BWS, SRS, TNDM, and PHP1B, but not in AS and PWS [22]. Two studies have used the HM450k array to quantify genome-wide DNA methylation for BWS and TNDM [28] or SRS patients [29] and concluded that multiple and even novel imprinted genes were abnormally methylated in IDs.

Here, we have studied DNA methylation at about 485,000 CpGs genome-wide in 24 PHP patients with proven but different types of *GNAS* epimutations. The methylomes analyzed for 58 imprinted DMRs revealed in addition to the expected *GNAS* methylation defect, also a multi-locus methylation abnormality in 9 of the 24 patients for 19 other imprinted DMRs that were not previously known for these PHP cases. However, patients did not share a common pattern for these multi-locus methylation abnormalities, and there was a high variability in the degree of methylation difference and the number of amplicons per patient that were abnormally methylated. A validation study with the EpiTYPER for the most common abnormally methylated DMRs *FANCC* and *SVOPL* indeed showed significant methylation differences in a replication cohort of PHP patients. The *FANCC* and *SVOPL* DMRs are not well studied, and further studies are needed to define the functional relevance of these methylation changes. Though larger cohorts are needed to be conclusive, based on results from both the HM450k and the EpiTYPER study, it seems that PHP^neg^ patients are more sensitive to these multi-locus defects compared to PHP^Δstx16^ patients. Other studies that have used targeted approaches with a limited number of known imprinted DMRs have also reported multi-locus defects in PHP patients. Perez-Nanclares et al. reported the first two PHP patients with multi-locus hypomethylation; one patient at *PEG1/MEST* and the other at *GTL2* [24]. Court et al. identified five PHP patients with hypomethylation at other imprinted loci: *PEG1/MEST*, *MCTS2*, *IGF2R*, *ZNF331*, *L3MTBL1*, and *MEG3* [22]. Maupetit-Méhouas et al. studied a larger cohort of 63 PHP patients for the methylation pattern at eight imprinted loci and found multi-locus imprinting defects for 4 PHP patients at *PEG1/MEST*, *L3MBTL1*, and *DLK1/GTL2* [23]. Though in our PHP cohort we observe a higher rate of multi-locus methylation defects (38 % of all patients), this is probably due to the fact that more DMRs were studied, but remarkably, most of these previously reported abnormal DMRs are also abnormal in our study. Docherty et al. identified novel candidate imprinted genes in patients with imprinting disorders and multi-locus methylation defects [28]; we identified hypomethylation of three candidate regions (*PPIEL*:Ex1-DMR, *WRB*:alt-TSS-DMR, and *NHP2L1*:alt-TSS-DMR) from this study in our patients with PHP1B. The clinical relevance of these regions has not yet been determined. It is possible that these genes are common in most imprinting disorders. Interestingly, we identified two patients with methylation changes in the *SNRPN* DMRs. These two patients also have hypomethylation of the novel candidate DMR *LOC100130522/PARD6G-AS1* [28]. Methylation changes in *SNRPN* DMRs are associated with PWS and AS, but these patients are very infrequent and moreover loss of methylation of the *SNRPN* DMR is rarely described in other imprinting disorders with multi-locus methylation disturbances. It has only been observed in two patients with SRS [30], two patients with BWS [22, 28], and one patient with TNDM [28]. In accordance with previous studies, our PHP patients with multi-locus methylation defects do not show evidence for phenotype differences with patients having only a *GNAS* methylation defect. Further studies including biochemical analyses are necessary to confirm these findings. The patient with the highest number of genes presenting with a methylation defect has severe hypertension with organ damage but a link with the methylation defect is not obvious. The other patients with multi-locus methylation defects have no additional clinical or molecular features. We identified five patients with simultaneous hypo- and hypermethylation. This finding is consistent with the report of Maupetit-Méhouas [23]. The epidominance hypothesis suggests that the phenotype is determined by the most strongly affected imprinted locus [23]. Patients with hypomethylation of multiple imprinted loci have been associated with increased frequency of developmental delay and congenital anomalies [31], but based on our current study, we have no evidence for a clinical impact. Of particular interest is the fact that methylation disturbances at multiple imprinted loci are now described in a subset of patients affected with different IDs, and this could point to a common pathogenic mechanism for IDs [32].

According to the original classification for PHP, we have included PHP1B patients with A/B only and partial or full broad methylation defects and PHP1A patients with a broad partial methylation defect. During the last years, it became clear for reasons specified in the introduction that this classification is no longer accurate. All CpGs from the genome-wide data within the *GNAS* cluster were analyzed using unsupervised hierarchical cluster analysis. This analysis divided our patients in two groups seemingly only dependent on having the *STX16* deletion or not (referred to as PHP^Δstx16^ and PHP^neg^). The studied intervals cover the 4 DMRs in *GNAS* but these loci are much larger compared to the amplicons that were previously studied by the different labs that have used MS-MLPA, Sequenom EpiTYPER or pyrosequencing (for details see Fig. 1). PHP1B patients 23 and 24 that were diagnosed by MS-MLPA and pyrosequencing having a partial broad GNAS methylation defect are now clustered more closer to the control group. The most intriguing finding is however the discovery of a novel previously not described DMR (named *GNAS-AS2*) in the *GNAS* cluster that was found hypomethylated for both PHP^Δstx16^ and PHP^neg^ patients. *GNAS-AS2* is located within the large *GNAS-AS1* region and is actually located in the promoter region of the *XL* exon 1 (Additional file 2: Figure S1) but is separated from *XL* by a hypermethylated region. Many studies have described that PHP1B patients with *STX16* deletion only have hypomethylation of the *A/B* DMR while the DMRs *XL*, *GNAS-AS1*, and *NESP* are normal [10–13]. This is also the case for the present HM450k data except for the CpGs that are located in the *GNAS-AS2* region. *GNAS-AS2* hypomethylation for both PHP^Δstx16^ and PHP^neg^ patients was validated with the EpiTYPER. Only 1/24 (1 with partial PHP1B—broad) and 4/42 PHP (3 with PHP1B—A/B only and 1 with partial PHP1B—broad) patients had a normal *GNAS-AS2* methylation based on HM450k and EpiTYPER data, respectively. Methylation defects affecting the maternal *A/B* DMR lead to the loss of Gsα expression in renal proximal tubules and renal PTH resistance. It is however not clear at this moment whether hypomethylation of the *GNAS-AS2* DMR has a phenotype impact or whether methylation studies of this region can be useful for a better characterization of PHP patients. Further experiments are also needed to determine if loss of methylation of DMR *GNAS-AS2* influences the expression of *GNAS-XL*.

## Conclusions

We here present the first genome-wide methylation study in different types of PHP. It seems that only the presence of the *STX16* deletion is of significant importance to divide PHP in two groups of patients with significantly different methylation profiles. Methylation changes in other imprinted DMRs seem to be enriched in especially PHP^neg^ patients. We identified a novel *GNAS*-*AS2* DMR in the *GNAS* locus that is hypomethylated in all PHP patients, independently from *STX16* deletion. Further studies must be undertaken to unravel the function and clinical impact of methylation changes in the *GNAS*-*AS2* region that in addition to the *A*/*B* DMR can also be hypomethylation in PHP patients with *STX16* deletion.

## Methods

### Patient samples

A total of 61 PHP patients were enrolled by 5 different endocrinology centers from the European PHP Consortium. The molecular diagnosis for PHP was performed by each center using the methylation detection assay for the *GNAS* DMRs as previously described with methodologies and the location of the studied amplicons in Fig. 1 and Table 1 [21]. All PHP patients included in this study have PTH resistance and were labeled with PHP1A if an obvious AHO phenotype was present (only brachydactyly and subcutaneous ossifications were noticed, Table 1). Based on the genetic screening for *STX16* deletions and *GNAS* methylation screening, laboratories were asked to label the patients with the following: (i) *STX16* deletion present or not, (ii) AB-only or broad methylation defect, and (iii) full or partial methylation defect [21]. This resulted in the use of the following labels (Table 1): PHP1B—A/B only, full PHP1B—broad, partial PHP1B—broad and partial PHP1A—broad. These terms are based on the original classification used for patients with PHP type I.

Table 1 presents the patients for the genome-wide study and Additional file 1: Table S2 the patients for the replication studies. The order of the patients in Table 1 was determined by unsupervised cluster analysis of genome-wide data for only the *GNAS* locus. The total number of PHP patients that were enrolled for the HM450k array and Sequenom studies are specified in Additional file 1: Table S2. Shortly, the genome-wide DNA methylation study was performed for 24 PHP patients and 20 age- and gender-matched healthy controls (Table 1). Validation of *NESP-AS2* was performed for 42 PHP patients and 20 controls (Additional file 1: Table S3). Validation of the methylation levels of *FANCC*, *SVOPL*, and *WDR27* was performed in 26 PHP patients and 12 controls (Additional file 1: Table S6).

Informed consent for methylation and genetic studies was obtained from all participants and/or their legal representatives after approval of these studies by local Ethical Committees.

### Genome-wide methylation profiling using the HM450k array

Genomic DNA was extracted from leukocytes using standard techniques and bisulfite converted using the EZ DNA methylation kit (Zymo Research, Irvine CA) as previously described [33]. The array was performed by the Genome Centre (Bart’s and the London School of Medicine and Dentistry, Londen, UK) on the HumanMethylation 450K BeadChip (Illumina) using manufacturer’s reagents and protocols. An identical control sample was assigned to each batch and samples were distributed randomly to control for batch effects. The correlation for the internal quality control was high (>0.99). The methylation level (*β* value) was calculated using the Methylation Module of BeadStudio software.

### HM450k data filtering and genome-wide analysis

After normalization of the data using GenomeStudio software, data were analyzed using a pipeline developed within the R statistical analysis environment (http://www.r-project.org, Bioconductor, Seattle, USA). Before analyzing the data, we excluded possible sources of technical bias. Probes with a high detection value (*p* > 0.01) in more than 10 % of the samples (644 probes) and probes containing any missing values (13,194 probes) were removed, as well as non-CpG and gender-matched probes. Finally, we excluded probes as they contained SNPs present in >1 % of the population and leukocyte-specific probes [34, 35]. In total, we analyzed 355,105 probes for all DNA samples (73 % of probes). No statistical batch control was required as all the cases and controls had been processed in the same time, and the correlation for the quality control was high (>0.99). The mean methylation and standard deviation were determined for the control population and individual samples. We did not detect any differentially methylated DMRs between the 20 control samples. DNA samples from imprinting syndrome patients were considered epimutated if the methylation value for an imprinted DMR differed was outside 3 standard deviations from the 20 control samples. Cluster analysis was performed within the R statistical analysis environment.

### HM450k data filtering for analysis *of* imprinted genes

We analyzed 58 imprinted DMRs that also included the DMRs of the *GNAS* cluster (Additional file 1: Table S4). The list was based on the known imprinted DMRs from the consensus list from the European Network of Human Congenital Imprinting Disorders (http://www.imprinting-disorders.eu). In addition, we included novel human imprinted DMRs from recent research that combined whole-genome bisulfite sequencing with HM450k to generate methylation profiles [36] as well as four novel candidate DMRs (*GLP2R*, *JAKMIP1*, *LOC100130522/PARD6G-AS1*, *SVOPL*) from research using genome-wide methylation profiling with HM450k in patients with multi-locus methylation defects [28]. These regions have not yet been further characterized to determine SNP versus parental-origin methylation and tissue profile. Some DMRs of the list are secondary DMRs (i.e., *DIRAS3*:Ex2-DMR, all the *SNRPN* DMRs).

### Validation of NESP-AS2 and FANCC, SVOPL, WDR27 methylation using the Sequenom EpiTYPER

Leukocyte DNA (1 μg) was subjected to bisulfite treatment using the MethylDetector™ bisulfite modification kit (Active Motif, Carlsbad CA, USA) as we described [21, 33, 36–38]. The Sequenom MassARRAY (Sequenom, San Diego, CA, USA) was used for quantitative DNA methylation analysis of the CpGs within the amplicons of *FANCC*, *SVOPL*, and *WDR27* using conditions described. Long cycling incubation was applied to further optimize the conversion reaction [39]. Primers were designed using the Sequenom EpiDesigner BETA software (www.epidesigner.com), taking into account amplicon coverage, number of CpGs, fragment size and number of nucleotide repeats in the primer sequence (Additional file 1: Table S7). PCR steps were performed in triplicate for each DNA sample and a standard deviation between replicates was mostly <10 %. When triplicate measurements had a SD >10 % or when only one of the triplicates was available, data for that sample were excluded. The mean of three values was used for further analyses. The EpiTYPER analysis method reports CpG methylation values as percentage. Statistical analyses to quantify DNA methylation differences were performed using the Prism 6 software (GraphPad Software Inc., San Diego). DNA samples from PHP patients were considered epimutated if the methylation value for an imprinted DMR was outside two and three standard deviations determined from the control samples. A two-tailed *T* test was used to assess differences in mean DNA methylation levels between cohorts for the overall amplicon considered as methylation average and for each CpG unit within this amplicon separately.

### GNAS molecular analysis

Genomic DNA was isolated from leukocytes. Genotype for the GNAS exon 5 SNP (rs7121) at codon 131 was determined by PCR with the forward primer 5′-ttggtagcgccctcccaggc-3′ and the reverse primer 5′- catgttcctatatggacactg-3′. After denaturation at 95 °C, 40 cycles of DNA amplification were performed using Taq PCR Mastermix at 95 °C for 30 s, 58 °C for 60 s, and 72 °C for 60 s. The PCR products were digested using the restriction enzyme FokI and analyzed on a 2 % agarose gel. Only samples heterozygous at *FokI* polymorphism were selected. One patient and one control were heterozygous. Total RNA was extracted from platelets using TRIzol (Invitrogen) reagent, according to the manufacturer’s protocol. The *GNAS* gene was amplified from platelet RNA to check expression of *XL*.

## Additional files

Additional file 1:
**This contains additional files for Tables S1-S7.**
Additional file 2:
**Detailed schematic representation of the human GNAS-AS region.**

## Abbreviations

A/B: exon A/B = *GNAS-A/B*:TSS DMR
AHO: albright hereditary Osteodystrophy
AS1: *GNAS* antisense = *GNAS-AS1*:TSS DMR
BWS: Beckwith-Wiedemann syndrome
DMRs: differentially methylated regions
HM450k: HumanMethylation 450K BeadChip
NESP: neuroendocrine secretory protein 55 = *GNAS-NESP*:TSS DMR
PHP: pseudohypoparathyroidism
PPHP: pseudopseudohypoparathyroidism
PTH: parathyroid hormone
SRS: Silver-Russell syndrome
TNDM: transient neonatal diabetes mellitus
TSH: thyroid-stimulating hormone
XL: extra-large stimulatory G protein = *GNAS-XL*:Ex1 DMR
