# Supplementary material for: Genome-wide DNA methylation analysis of pseudohypoparathyroidism patients with GNAS imprinting defects
Source: Clin Epigenetics. 2016 Jan 26;8:10. doi: 10.1186/s13148-016-0175-8 (PMC4728790; doi:10.1186/s13148-016-0175-8)
Supplement: Additional file 2: — Detailed schematic representation of the human GNAS-AS region. [file 13148_2016_175_MOESM2_ESM.docx]

**Figure S1. Detailed schematic representation of the human GNAS-AS region.**

**
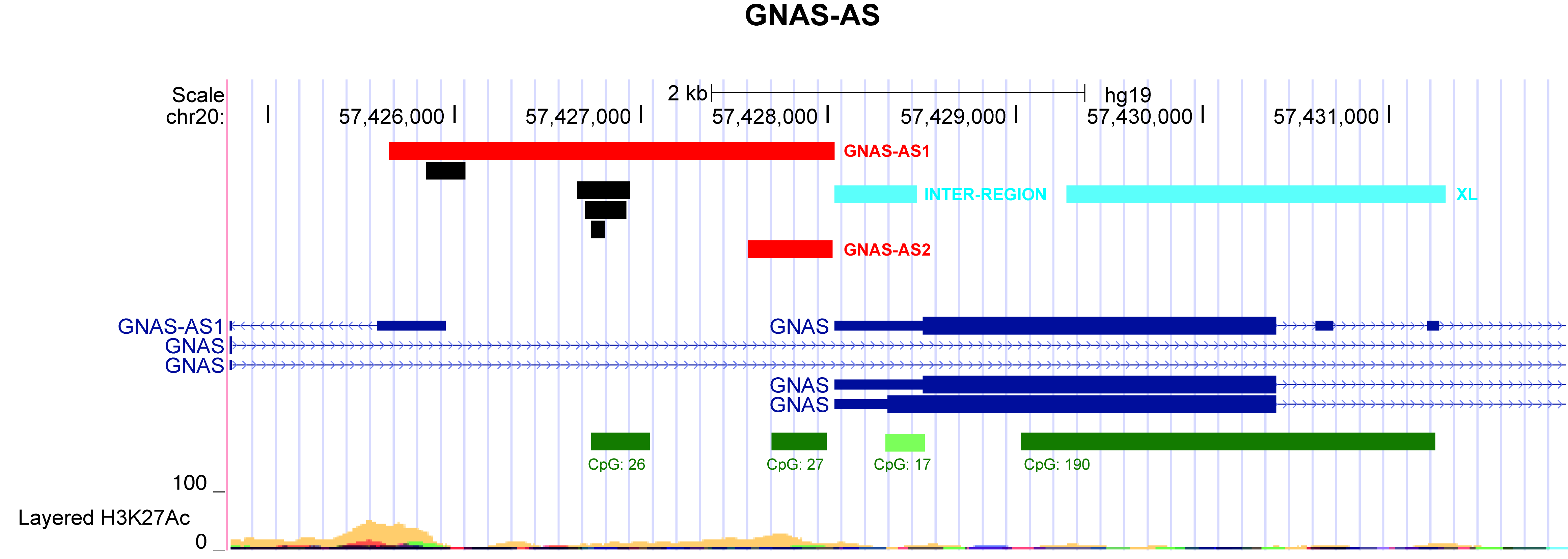
**

Bars indicate the different *GNAS-AS* methylation studies that were performed in this study using the 450K BeadChip (GNAS-AS1) and Sequenom EpiTYPER (GNAS-AS2) and previously, by the different European centers (black bars). Nucleotide positions accord to NCBI build 37/hg19.
